# Classification of pleasantness of wind by electroencephalography

**DOI:** 10.1371/journal.pone.0299036

**Published:** 2024-02-27

**Authors:** Yasuhisa Maruyama, Ryuto Nakamura, Shota Tsuji, Yingli Xuan, Kunio Mizutani, Tsubasa Okaze, Natsue Yoshimura

**Affiliations:** 1 School of Computing, Tokyo Institute of Technology, Yokohama, Japan; 2 School of Environment and Society, Tokyo Institute of Technology, Yokohama, Japan; 3 School of Engineering, Tokyo Institute of Technology, Yokohama, Japan; 4 Faculty of Engineering, Tokyo Polytechnic University, Atsugi, Japan; 5 ATR Brain Information Communication Research Laboratory Group, Kyoto, Japan; Shahid Beheshti University, ISLAMIC REPUBLIC OF IRAN

## Abstract

Thermal comfort of humans depends on the surrounding environment and affects their productivity. Several environmental factors, such as air temperature, relative humidity, wind or airflow, and radiation, have considerable influence on the thermal comfort or pleasantness; hence, these are generally controlled by electrical devices. Lately, the development of objective measurement methods for thermal comfort or pleasantness using physiological signals is receiving attention to realize a personalized comfortable environment through the automatic control of electrical devices. In this study, we focused on electroencephalography (EEG) and investigated whether EEG signals contain information related to the pleasantness of ambient airflow reproducing natural wind fluctuations using machine learning methods. In a hot and humid artificial climate chamber, we measured EEG signals while the participants were exposed to airflow at four different velocities. Based on the reported pleasantness levels, we performed within-participant classification from the source activity of the EEG and obtained a classification accuracy higher than the chance level using both linear and nonlinear support vector machine classifiers as well as an artificial neural network. The results of this study showed that EEG is useful in identifying people’s transient pleasantness when exposed to wind.

## Introduction

Our daily productivity is largely influenced by the thermal comfort of our environment, and prolonged exposure to an uncomfortable environment can often be harmful to our health [1]. Especially, air temperature and relative humidity greatly affect our comfort levels; hence, they are usually controlled with air-conditioning systems in indoor spaces. Airflow (or wind) is used to increase the efficiency of thermal control and is highly effective in that it can provide instantaneous pleasantness. Moreover, since wind can be delivered promptly, it is considered a very important factor for achieving and maintaining an optimal environment immediately [2] and with high energy efficiency [3]. Natural ventilation systems also have the potential for improvement of thermal comfort with energy-saving even though comprehensive control is more effective depending on the outside weather condition [4]. Accordingly, algorithms for the automatic control of electrical devices have been studied to realize optimal environments without manual control. However, there is a growing need for objective measurement methods for thermal comfort and pleasantness.

One of the widely recognized indices to quantify thermal comfort is the predicted mean vote (PMV) [5]. PMV estimates how hot or cold people feel in the environment based on the air temperature, humidity, air velocity, mean radiant temperature, clothing, and metabolic rate. However, because the PMV estimates the heat balance under steady or quasi-steady states, it is difficult to consider people’s instantaneous physiological and psychological states, which are transient. Accordingly, other measures need to be employed to recognize personal transient thermal comfort to achieve and maintain personally optimized and energy-efficient environments.

One solution is to use physiological signals, such as electroencephalography (EEG), skin temperature, galvanic skin response, and heart rate [6, 7]. For example, Yao et al. investigated the relationship between thermal comfort and physiological signals, such as EEG, skin temperature, and electrocardiogram, by manipulating the ambient temperature [8, 9]. Some other studies have predicted thermal comfort levels based on physiological signals using machine learning methods [10–13]. Wu et al. performed classification analyses between comfortable and uncomfortable states using EEG [10]. They also performed an online experiment to control an air conditioner based on participants’ thermal comfort predicted using their EEG [11]. In addition, Shan et al. and Shan and Yang used machine learning methods to distinguish between several thermal conditions [12, 13]. These studies focused on EEG because it directly measures the brain activity, which may reflect the personal thermal comfort.

Although wind can provide instantaneous pleasantness, most previous studies have focused on the relationship between EEG and ambient temperature and/or relative humidity, and only a few studies have investigated the relationship between EEG and airflow. Okamoto et al. and Tamura et al. performed indoor experiments and reported that beta and gamma bands of EEG showed different activities between different airflow conditions and were related to comfort levels [14–16]. Furthermore, Raheel et al. utilized hot and cold air to increase the emotional experience in tactile-enhanced multimedia and reported improved prediction accuracy in EEG-based emotion recognition compared with no-airflow conditions [17]. Although these studies showed that some EEG characteristics reflect the emotional components related to airflow, it remains unclear whether EEG can be used to recognize the instantaneous pleasantness of fluctuations in airflow.

**Fig 1:**
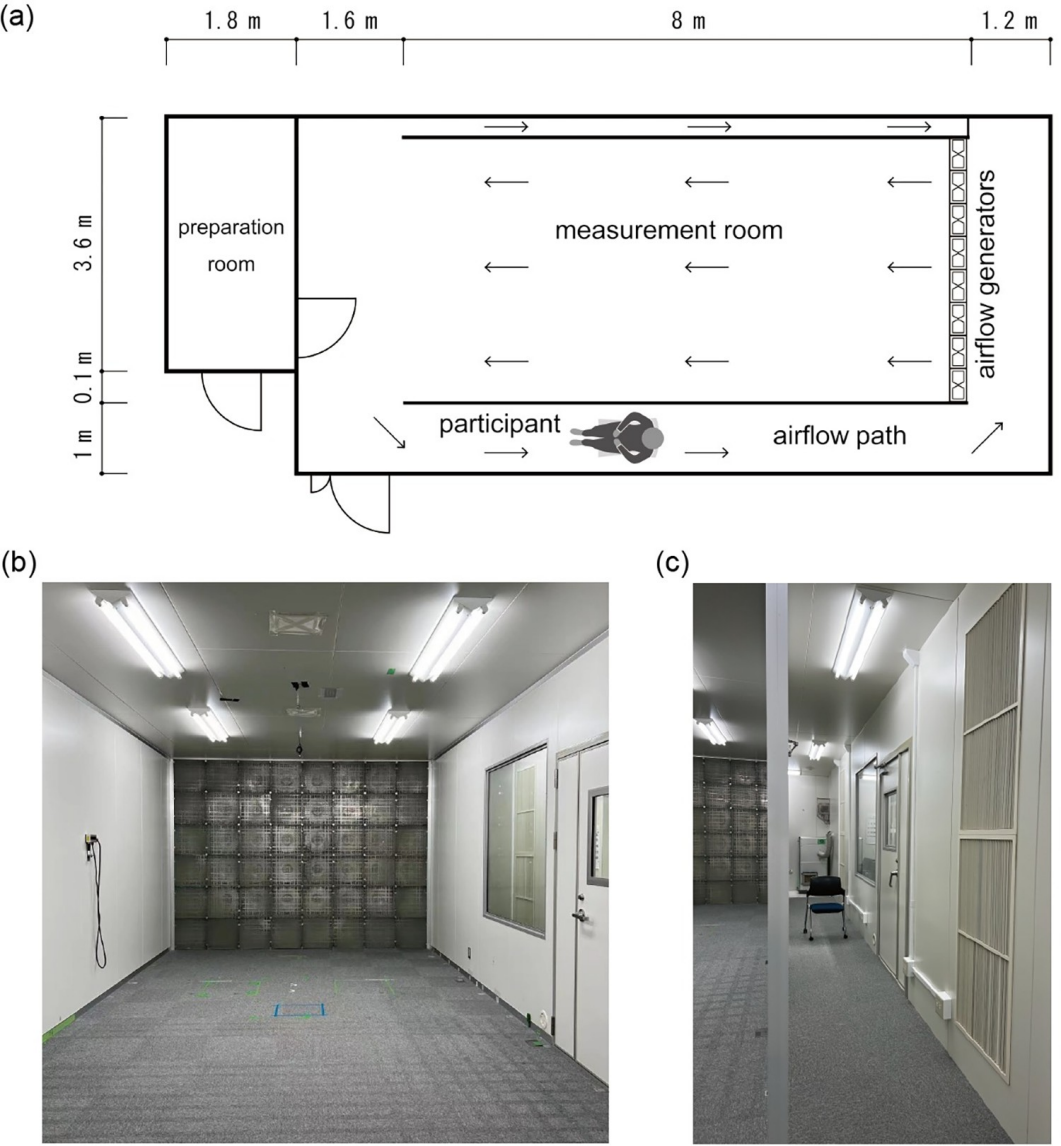
Design of the artificial climate chamber. (**a**) Floor plan. Arrows indicate airflow (wind). (**b**) Internal view of the measurement room. This photograph was captured in the preparation room. Airflow generators (electric fans) are installed on a wall. (**c**) Internal view of the airflow path. This photograph was captured from around the preparation room. Participants sat on a chair placed in the middle of the path.

Accordingly, the present study investigated whether EEG is useful in recognizing the instantaneous pleasantness of wind. If the pleasantness of wind that people were exposed to can be recognized through EEG, it would lead to the realization of an optimal and energy-efficient environment through automatic control of electrical devices producing airflow in the future. We conducted an experiment in an artificial climate chamber where wind could be generated (Fig 1) and performed an EEG-based classification of the pleasantness of the ambient airflow reproducing the natural wind fluctuations using machine learning techniques. Participants experienced ambient airflows of one of four velocity levels on their anterior side for 10 s and reported their pleasantness levels after the wind stopped. Because the brain regions that show activities related to the instantaneous pleasantness of thermal stimuli were identified in a previous functional magnetic resonance imaging (fMRI) study [18], we estimated the source-level activity from the channel EEG, and only the signals in those brain regions were used in the classification analysis. Comparison between different machine learning methods was also performed in this study.

## Materials and methods

### Participants

Participants were recruited from the Tokyo Institute of Technology and Tokyo Polytechnic University. Eighteen healthy undergraduate and graduate students between 21 and 30 years of age (8 females, 10 males; mean age (±standard deviation), 23.5 (±2.0) years) participated in this study. All participants were right-handed and had no history of neurological or psychiatric disorders. Prior to the experiment, all participants provided written informed consent. This study was approved by the Institutional Review Boards of the Tokyo Institute of Technology (Approval No. 2018170) and Tokyo Polytechnic University (Approval No. 2019–05), and the experiment was conducted in accordance with the Declaration of Helsinki.

### Experiment

The experiment was performed in an artificial climate chamber at the Atsugi Campus of Tokyo Polytechnic University (Fig 1) in September 2019. During the experiment, the air temperature and relative humidity were maintained at 30°C and 70%, respectively. The chamber was equipped with 48 electric fans that produced wind on a wall, and the wind velocity could be controlled by a computer. During the experiment, the participants sat on a chair in the airflow path such that they could not see the fans. To adjust their body to hot and humid conditions, the participants entered the chamber approximately 1 h before the experiment commenced, during which time EEG electrodes and skin temperature sensors were attached.

**Fig 2:**
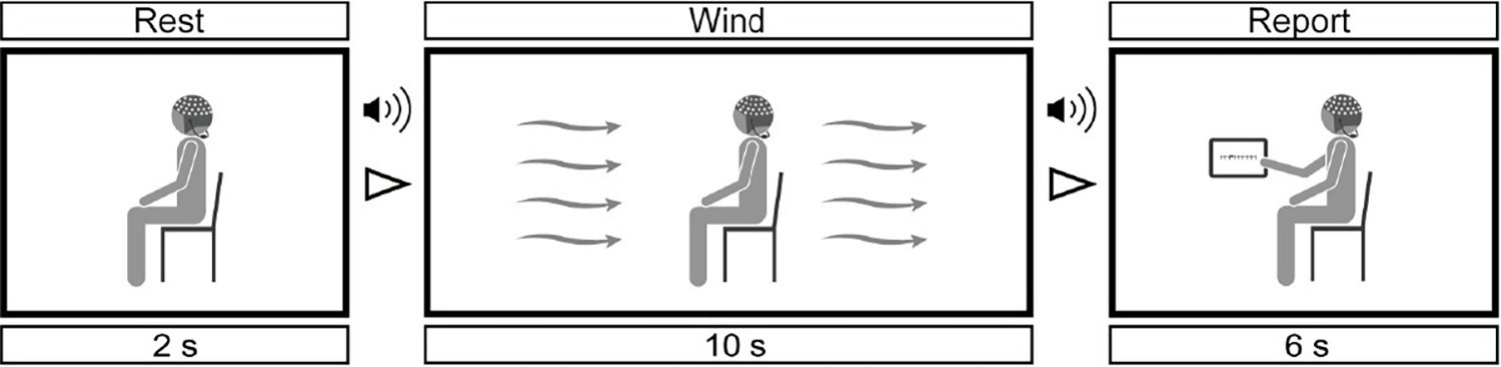
Trial flow. During 10 s of wind-blowing period, participants were exposed to airflows with velocities of 0.44, 1.0, 2.0, and 4.0 m/s. During 6 s of the pleasantness-reporting period, the participants reported their pleasantness using a computerized visual analog scale. The participants wore noise-canceling earbuds throughout the experiment, and the start and end of the wind-blowing periods were notified to them through the earbuds. The experiment consisted of 120 trials.

The flow of the trial in the experiment is shown in Fig 2. One trial consisted of a rest period of 2 s, a wind-blowing period of 10 s, and a pleasantness-reporting period of 6 s. During the wind-blowing period, the participants kept their eyes closed, and the wind blew onto the anterior side of the participants. To eliminate the sound produced by the fans, the participants wore noise-canceling earbuds through which pink noise was played continuously during the experiment. The start and end of the wind-blowing periods were notified to the participants by a beep sound transmitted through the earbuds. After the beep sound denoting the end of the wind-blowing period, the participants opened their eyes and reported their pleasantness using a computerized visual analog scale ranging from 1.0 to 9.0 in 0.1-point steps. Values of 1.0, 5.0, and 9.0 represented unpleasant, neutral, and pleasant states, respectively. Each session consisted of 40 trials, and each participant completed three sessions (120 trials in total). In this study, four wind velocity levels were used to induce different levels of pleasantness. In particular, participants were exposed to winds of 0.44, 1.0, 2.0, and 4.0 m/s measured at the participant seat located at 1.1 m height in the trial. The thermal sensation of participants with PMV was calculated to be slightly warm when the participants were exposed to wind speeds of 1.0 or 2.0 m/s at an air temperature of 30°C and relative humidity of 70%, assuming the outside environment in the summer of Tokyo, for enough a long time. Then, almost half of the 1.0 m/s and twice of 2.0 m/s wind speed conditions were introduced. The turbulence intensity of the exposed wind was approximately 30% for all wind velocities to reproduce the natural wind velocity fluctuations in an outdoor environment by devising a flow channel in the chamber. The wind velocity was presented in a pseudo-random order such that the same wind velocity was not assigned to two or more consecutive trials.

EEG data were recorded with 64 electrodes using eego sports 64 (ANT Neuro b.v., Hengelo, The Netherlands) at a sampling frequency of 512 Hz. EEG electrodes were placed according to the 10/10 system. The skin temperature was measured at the left chest, upper arm, thigh, and lower leg during the experiment by using a Z2015 heat flow sensor (HIOKI E.E. CORPORATION, Nagano, Japan) at a sampling frequency of 0.1 Hz, although these temperature data were not analyzed in the current study.

### EEG processing

We preprocessed the EEG data using EEGLAB version 2020 [19] and MATLAB R2020b (MathWorks, Inc., Natick, MA, USA). After applying a high-pass filter (0.5 Hz) and a low-pass filter (45 Hz) to remove low-frequency drift and high-frequency noise, we extracted the epochs consisting of 1 s of pre-wind period and 10 s of wind-blowing period from the continuous EEG data. We then removed noisy channels (mean across participants: 1.0), interpolated them using the spherical spline method, and rejected epochs containing large artifacts, such as muscle activity (mean across participants: 4.2), by visual inspection. Subsequently, independent component analysis was applied to the EEG data, and the independent components that appeared to represent activities related to eye movements were removed. The EEG data were then re-referenced to the average of all EEG channels. We excluded the first 2 s of the wind-blowing period in each trial; thus, we analyzed only the periods during which the wind velocity was stable.

To minimize the influence of other neural activities in the brain on the classification of the pleasantness of wind, the pleasantness-related signals were extracted and used as the features for classification in this study. Based on a previous fMRI study that identified brain regions showing activities related to the instantaneous pleasantness of thermal stimuli [18], we estimated the source-level activity from the channel EEG, and only signals from those brain regions were included in the analysis. The estimation of the source activity from the channel EEG data was performed using the exact low-resolution brain electromagnetic tomography (eLORETA) method [20–22] after baseline correction of the channel EEG on MNE-Python [23, 24]. eLORETA is a linear imaging method for estimating the distributed source activity and achieves zero localization error. The noise covariance matrix was calculated using 1 s of pre-wind periods. We estimated the source activity at 4098 vertices per hemisphere in the cerebral cortex of the template brain. The orientation of each dipole was fixed perpendicularly to the cortical surface.

The power spectral density was calculated using Welch’s method for each vertex [25]. The window length and overlapping segments were 512 (1 s) and 256 (0.5 s), respectively, and the Hann window was applied as the window function. To reduce the computational cost, the vertices were assigned to 68 brain regions based on the Desikan–Killiany atlas [26], and the power at all the corresponding vertices was averaged for each brain region. Subsequently, the powers of the theta (4–7 Hz), alpha (8–13 Hz), beta (14–30 Hz), and gamma (31–45 Hz) bands were calculated by averaging the powers at the corresponding frequency bins.

To reduce the feature dimension and prevent irrelevant brain activity from affecting the classification, the brain regions in the cerebral cortex that were reported in a previous fMRI study related to the instantaneous pleasantness of thermal stimuli were used in the classification analysis [18]. Specifically, the rostral anterior cingulate cortex, medial orbitofrontal cortex, and lateral orbitofrontal cortex were included in both hemispheres.

### Classification analysis

We performed a within-participant binary classification of wind pleasantness based on the reported pleasantness scores. One class, labeled as the pleasantness class, included trials with reported pleasantness scores greater than 5.0, and the other class, which consisted of trials with reported pleasantness scores less than 5.0, was labeled as the unpleasantness class. Data from two participants (Participants 17 and 18), where either class had fewer than 30 trials, were excluded from the classification analysis. The power values of the four frequency bands in six regions (three regions per hemisphere) were used as the features for the classification analysis after logarithmic transformation.

In this study, we tested four classifiers: logistic regression, support vector machine (SVM) with the linear kernel, SVM with the radial basis function (RBF) kernel, and artificial neural network (ANN) with one hidden layer. In the ANN, AdamW was used as the optimizer [27], and the sigmoid function and rectified linear unit (ReLU) were used as the activation functions in the output and hidden layers, respectively. Logistic regression and SVM were performed using *the scikit-learn* package [28], and ANN was performed using *the TensorFlow* package [29] in Python.

**Fig 3:**
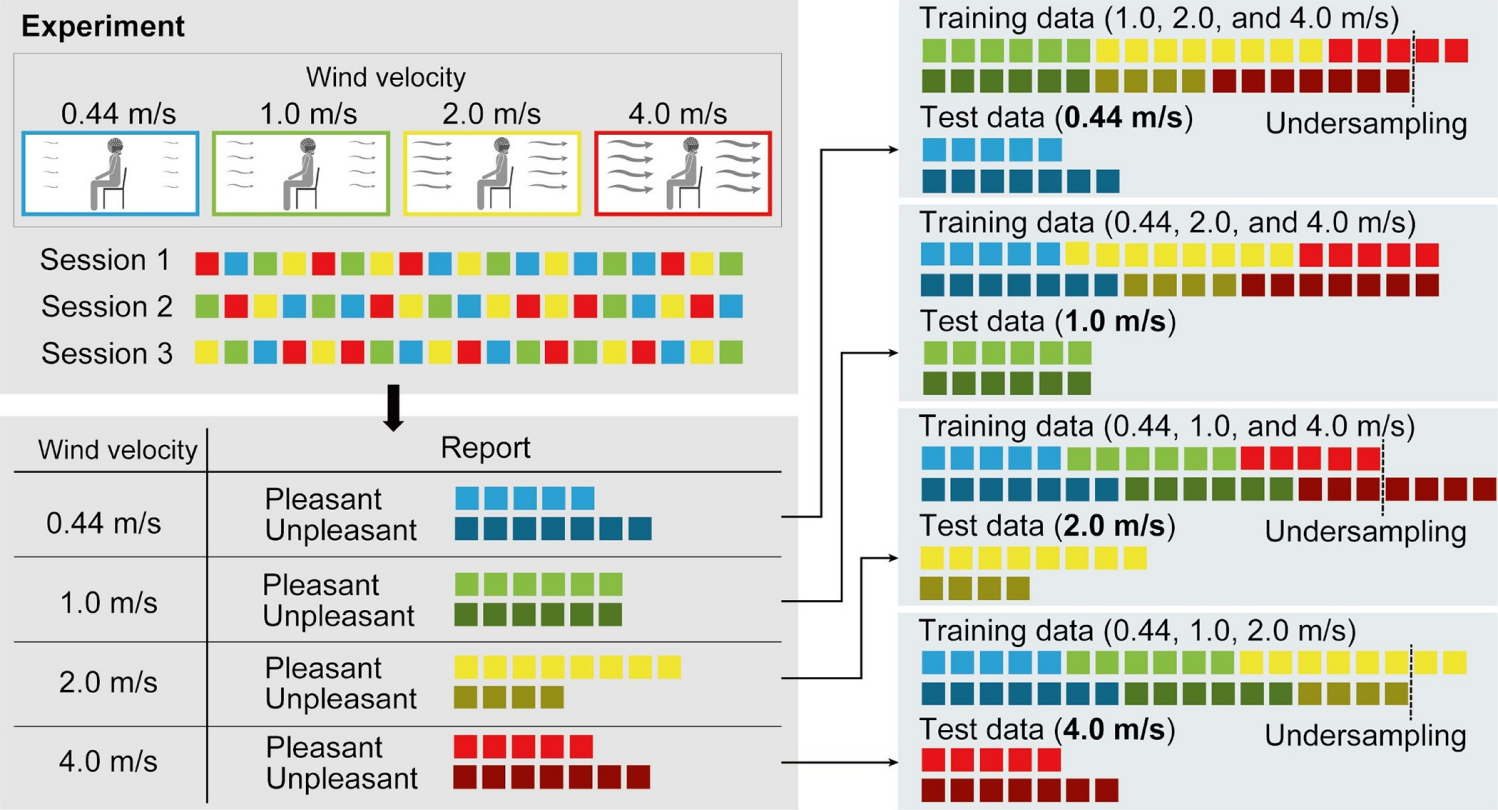
Schematic illustration of classification analysis. Training data consisted of trials of 3 out of 4 wind velocity conditions, and trials of the remaining wind velocity condition were used as test data. In the actual experiment, one session consisted of 40 trials, and in the analysis, trials to be excluded were randomly selected in the undersampling step.

To eliminate the effect of wind velocity on the classification of pleasantness, trials of the same wind velocity conditions were used only in either the training or test data in the classification analysis. Specifically, the classification accuracy was calculated by training the classifiers using trials from three out of four wind velocity conditions and testing them using trials from the remaining wind velocity condition. Fig 3 shows the schematic of this step. For example, when the test data consisted of trials with wind velocity of 0.44 m/s, the training data consisted of trials with wind velocities of 1.0, 2.0, and 4.0 m/s. This procedure was repeated four times such that each wind velocity condition was assigned to the test data once, and the mean accuracy was reported as the participant’s classification accuracy. We performed the analysis in this manner to prevent the classification accuracy from reflecting the wind velocity rather than pleasantness, because the wind velocity and pleasantness scores were closely related. We did not perform the classification when the data of either class contained less than 30 trials in the original training data, as a shortage of training data can lead to overfitting. Before training the classifiers, trials of a class with larger samples were randomly undersampled in the training data such that both classes had equal numbers of trials, and the classifiers were not biased to either class. We repeated these procedures five times and reported the mean classification accuracies. In each repetition, trials used for training the classifiers were randomly selected in the undersampling step so that the same dataset was not used for training more than once. Before training and testing the classifiers, all the features were normalized using the means and standard deviations of the undersampled training data.

Hyperparameters, including the regularization parameter in logistic regression; the regularization parameter and gamma in SVM; and the number of units in the hidden layer, batch size, learning rate, weight decay parameter, and number of epochs in ANN, were determined using the undersampled training data, where 20% of them were used as the validation data. Hyperparameter tuning was performed based on the tree-structured Parzen estimator algorithm [30] in *Optuna* software [31].

A one-tailed one-sample t-test was used to determine whether the classification accuracy of each classifier was significantly higher than the chance level (i.e., 0.5). Holm–Bonferroni correction was applied to the p-values to account for the multiple comparison problem [32]. In addition, we compared the classification accuracies of all four classifiers. One-way repeated-measures analysis of variance (ANOVA) was used to examine whether the classification accuracy was different among the classifiers, and a p-value with Greenhouse–Geisser correction was reported.

To investigate the frequency bands that largely contributed to the classification, we calculated the feature importance of each frequency band by calculating the decrease in accuracy when the features at the frequency band were removed. In this method, all the features in each frequency band were removed from both the training and test data, and the decrease in the classification accuracy relative to the baseline setting using all features was calculated. Because the feature set consisted of the log power values at four frequency bands in six brain regions, this procedure was repeated four times by altering the excluded frequency band. SVM with the RBF kernel had the best classification accuracy among all tested classifiers; hence, it was used to calculate the feature importance scores. This procedure was executed for each frequency band, and we reported the mean feature importance scores of the participants. To determine whether the feature importance score in each frequency band was significantly higher than 0, a one-tailed one-sample t-test was used and the p-values were corrected with Holm–Bonferroni method [32]. Moreover, the difference in the feature importance score among the frequency band was examined with one-way repeated-measures ANOVA.

## Results

### Ratings of pleasantness

**Fig 4:**
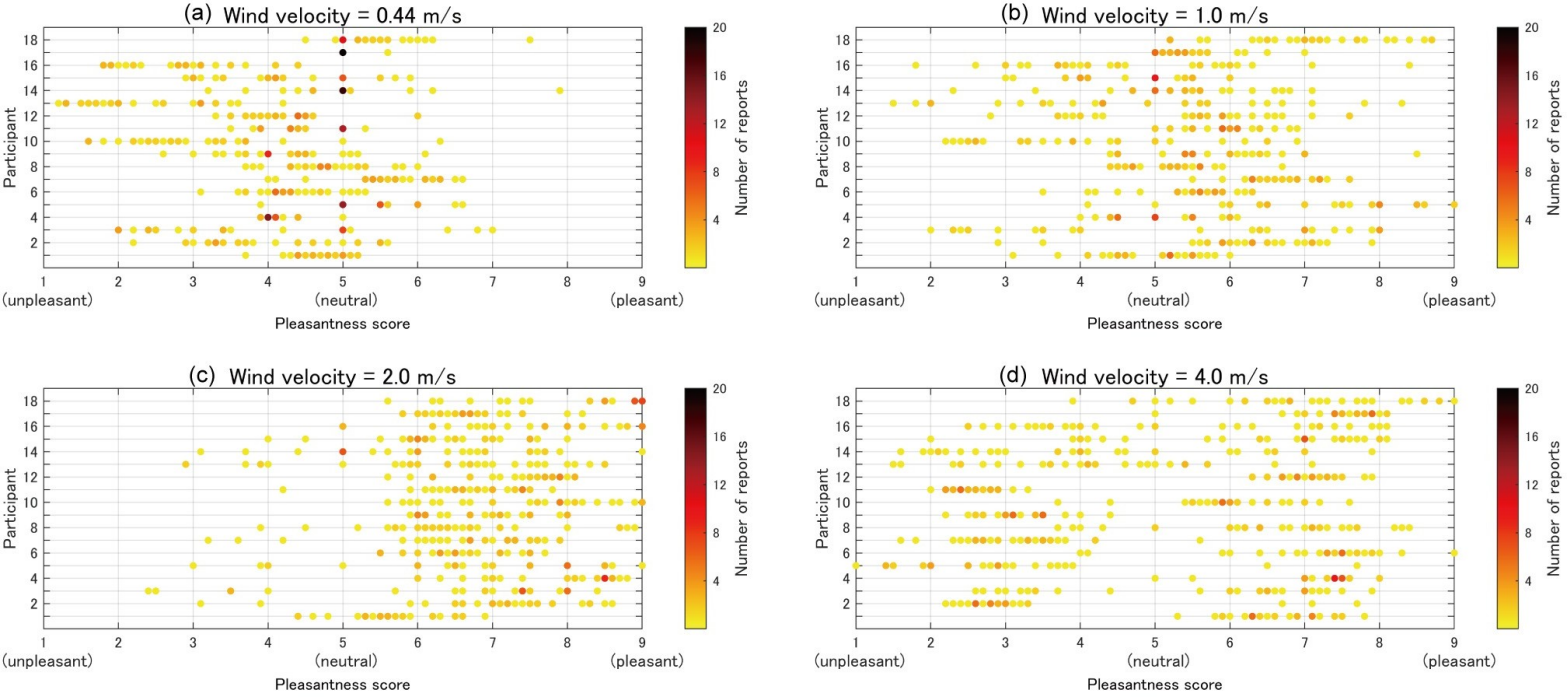
Distribution of reported pleasantness scores. Horizontal axis depicts the pleasantness scores, where values of 1.0, 5.0, and 9.0 denote unpleasant, neutral, and pleasant states, respectively. Vertical axis indicates each participant. The color of the dots represents the number of reports. Wind velocity of: (a) 0.44 m/s, (b) 1.0 m/s, (c) 2.0 m/s, and (d) 4.0 m/s.

Fig 4 shows the distribution of the reported pleasantness scores for all 18 participants under each wind velocity condition. The scores of 1.0, 5.0, and 9.0 represent unpleasant, neutral, and pleasant states, respectively. The mean reported pleasantness scores for the 18 participants were 4.3, 5.5, 6.9, and 5.3 during exposure to wind with velocities of 0.44, 1.0, 2.0, and 4.0 m/s, respectively. These results indicate that the participants found the second-strongest wind (2.0 m/s) most pleasant, while the weakest wind (0.44 m/s) induced unpleasantness in most participants. Because pleasantness scores for the strongest wind condition (4.0 m/s) were lower than those for the second- and third-strongest wind conditions (1.0 and 2.0 m/s), the pleasantness scores had a nonlinear relationship with the wind velocity.

### Classification

**Fig 5:**
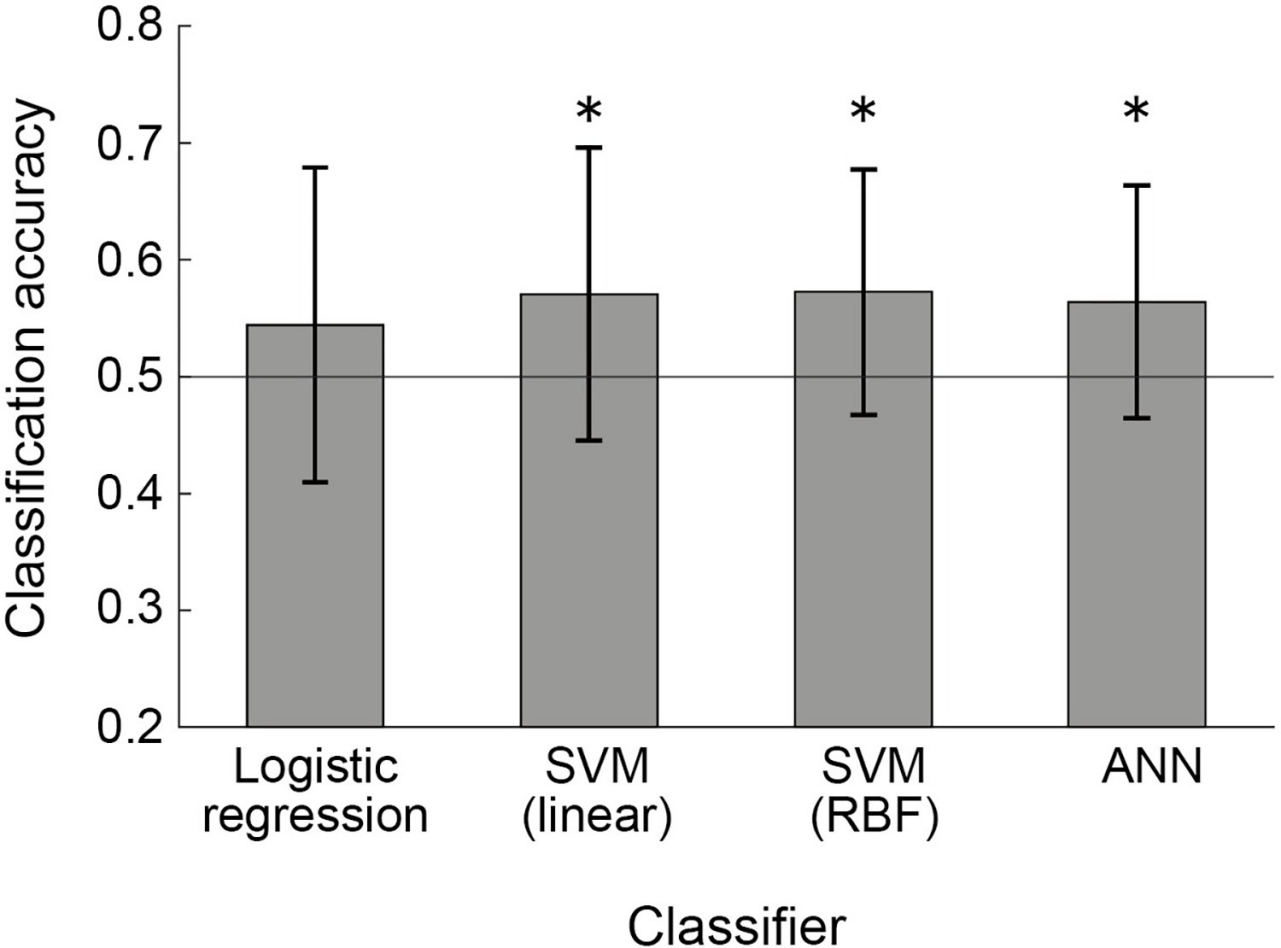
Mean classification accuracy of the binary classifiers. Error bars represent standard deviations. *p<0.05 in t-test with Holm–Bonferroni correction.

The mean classification accuracies of the binary classifiers are shown in Fig 5. The mean classification accuracies (±standard deviation) were 0.544 (±0.135), 0.571 (±0.125), 0.573 (±0.105), and 0.564 (±0.100) for logistic regression, SVM with the linear kernel, SVM with the RBF kernel, and ANN, respectively. SVM with the linear kernel, SVM with the RBF kernel, and ANN showed significantly higher classification accuracy than chance (p<0.05 in the one-sample t-test with Holm–Bonferroni correction [32]). Statistically significant differences in classification accuracy among the classifiers were not observed in the repeated-measures ANOVA (p = 0.39). Table 1 shows the feature importance scores for the four frequency bands. The SVM with the RBF kernel was used for the computation. None of the feature importance scores were significantly higher than 0 in any frequency band, and the scores did not exhibit differences among the frequency bands (p = 0.65 in repeated-measures ANOVA).

**Table 1:** Feature importance of each frequency band.

Frequency band	Theta	Alpha	Beta	Gamma
**Mean feature importance (standard deviation)**	0.013 (0.080)	0.024 (0.068)	0.004 (0.073)	−0.004 (0.057)

## Discussion

In this study, the participants were exposed to four wind velocities with turbulent fluctuations in a hot and humid room. Within-participant binary classification using their EEG and reported pleasantness scores achieved higher classification accuracy than chance when SVM or ANN was utilized. In the analysis, we eliminated the possibility of decoding the wind velocity as much as possible by using EEG signals solely from the instantaneous pleasantness-related brain regions in the frontal cortex and by assigning different wind velocity conditions for training and testing the classifiers. Source-level analysis enabled the selection of brain regions used for classification.

In an indoor experiment performed in an artificial climate chamber, we succeeded in changing the participants’ pleasantness by manipulating the airflow velocity within the room. Different wind velocity conditions successfully induced varying levels of pleasantness. Moreover, depending on their instantaneous physiological and psychological states, a wide range of pleasantness scores was reported by the same participant, even under the same wind velocity conditions. This result confirms that information about environmental conditions alone is insufficient to recognize the instantaneous pleasantness experienced by individuals, and physiological signals that reflect their emotions are helpful.

Brain regions associated with emotions tend to differ according to the stimulus modality of the emotion elicitation method [33]. To date, some fMRI and positron emission tomography studies have investigated the brain regions associated with thermal comfort or pleasantness when perceiving non-painful thermal stimuli presented on the hand or body [18, 34–38]. Among these studies, Rolls et al. investigated the brain regions related to the instantaneous pleasantness of thermal stimuli using fMRI [18]. They reported that activities in the mid-orbitofrontal cortex, pregenual cingulate cortex, and ventral striatum were positively correlated with the subjective ratings of instantaneous pleasantness of thermal stimuli presented on the hand, while unpleasantness was correlated with the activity in the lateral orbitofrontal cortex. They also reported that the somatosensory cortex and ventral posterior insula showed activities correlated with the intensity of the thermal stimuli, and thus suggested that affective values and sensory properties of thermal stimuli were processed in different brain regions [18]. Accordingly, to make the classification of pleasantness of wind robust and less affected by other neural activities in the brain, we utilized source localization methods to estimate the source-level activity from scalp EEG and extracted features from the rostral anterior cingulate cortex, medial orbitofrontal cortex, and lateral orbitofrontal cortex for the classification analysis based on the Desikan–Killiany atlas [26]. Because it is difficult to measure the subcortical activity using EEG, the ventral striatum was not included in the analysis. As a result, we successfully decoded the instantaneous pleasantness of wind using the source-level EEG signals in these brain regions, although signals from their surrounding regions may also have affected the results due to signal leakage and spread of information in the source space [39, 40]. The result of the feature importance scores calculated using SVM with the RBF kernel showed that each frequency band alone did not have a large influence on the decoding performance in this study. This indicates that power in the four frequency bands were correlated with one another. Accordingly, we can expect that the pleasantness of wind can be classified using less features, leading to reduced computational cost during training the recognition models.

In the analysis, we classified the pleasant and unpleasant states using the frequency-domain features of EEG, and the classification accuracies of both SVM and ANN were greater than the chance level with statistical significance. Because no statistical difference was observed among the classification accuracies of the machine learning methods utilized in this study, successful decoding of the pleasantness of wind from EEG may not be dependent on specific types of classifiers. As described earlier, we eliminated the influence of wind velocity on the classification accuracy. The test data consisted of trials from one wind velocity condition that was not included in the training data. Additionally, we excluded brain regions associated with somatosensory processing, such as the somatosensory and motor cortices, from the classification analysis. Thus, this study succeeded in decoding the pleasantness per se rather than decoding the tactile sensation (i.e., wind velocity).

Although we achieved a classification accuracy higher than the chance level in this study, there are several limitations that should be acknowledged:

The first limitation is the relatively long duration of the wind-blowing period in the experiment. Participants were exposed to the wind for 10 s in each trial. Future research should consider conducting EEG experiments with shorter wind-blowing periods to investigate whether pleasantness can be decoded in shorter time segments. In addition, continuous EEG experiments with varying wind strengths over time and frequent reports of pleasantness scores could help explore whether time-series changes in pleasantness can be tracked from EEG data.

The second limitation relates to the number of classes used in the classification analysis. In this study, we divided the pleasantness scores into pleasant and unpleasant classes, performing binary classification. Due to the shortage of trials in some classes when attempting to divide pleasantness scores into more than two classes, we could not increase the number of classes in the analysis. Further experiments that include classification between more than two classes or using regression analysis will be necessary to enable the decoding of pleasantness levels at a finer scale from EEG data.

The third limitation concerns the number of EEG electrodes. Although we used 64 electrodes in this study, it is preferable to use less electrodes for practical use. Accordingly, methods to decode pleasantness with a smaller number of EEG electrodes should be developed in future research.

The last limitation concerns the relatively low classification accuracy. The classification accuracy in this study (57.3% in binary classification) is less than that of existing studies. Wu et al. reported group-level classification accuracies of up to 87.9% and 74.4% in their binary classification analyses between thermally comfortable and uncomfortable conditions, respectively [10, 11]. One potential reason for the difference in classification accuracy could be the different environmental factors manipulated in the experiments; wind velocity was manipulated in our study, while Wu et al. manipulated air temperature [10, 11]. Moreover, due to the inherent connection between environmental factors and thermal comfort, it is possible that the environmental factor (i.e., air temperature) itself affected the brain activity rather than thermal comfort, and directly contributed to the classification performance in the previous studies. In this study, we confirmed that pleasantness-related neural activity is reflected in the EEG data even after eliminating the possible effect of environmental factors. However, achieving a much higher classification accuracy is necessary for practical applications. A possible solution is to combine several physiological signals. It has been shown that combining EEG with other peripheral physiological signals, such as galvanic skin response, heart rate, and heart rate variability, increases the performance of emotion recognition [41]. Another solution is to integrate physiological signals with existing measures of thermal comfort, such as the PMV. The PMV provides a rough estimate of the thermal comfort, and physiological signals can provide complementary information. To summarize, the existing measures of thermal comfort combined with several types of physiological signals can realize high-performance recognition of the pleasantness of wind to provide a personalized comfortable environment that leads to increased human productivity and well-being in addition to energy efficiency.

## References

[1] Al Horr Y, Arif M, Kaushik A, Mazroei A, Katafygiotou M, Elsarrag E. Occupant productivity and office indoor environment quality: A review of the literature. Build Environ. 2016;105:369–389. doi: 10.1016/j.buildenv.2016.06.001

[2] Akimoto T, Tanabe S, Yanai T, Sasaki M. Thermal comfort and productivity—Evaluation of workplace environment in a task conditioned office. Build Environ. 2010;45(1):45–50. doi: 10.1016/j.buildenv.2009.06.022

[3] Veselý M, Zeiler W. Personalized conditioning and its impact on thermal comfort and energy performance—A review. Renew Sustain Energy Rev. 2014;34:401–408. doi: 10.1016/j.rser.2014.03.024

[4] Zhang H, Yang D, Tam VWY, Tao Y, Zhang G, Setunge S, et al. A critical review of combined natural ventilation techniques in sustainable buildings. Renew Sustain Energy Rev. 2021;141:110795. doi: 10.1016/j.rser.2021.110795

[5] Fanger PO. Thermal comfort: Analysis and applications in environmental engineering. Copenhagen: Danish Technical Press; 1970.

[6] Mansi SA, Barone G, Forzano C, Pigliautile I, Ferrara M, Pisello AL, et al. Measuring human physiological indices for thermal comfort assessment through wearable devices: A review. Measurement. 2021;183:109872. doi: 10.1016/j.measurement.2021.109872

[7] Feng Y, Liu S, Wang J, Yang J, Jao Y-L, Wang N. Data-driven personal thermal comfort prediction: A literature review. Renew Sustain Energy Rev. 2022;161:112357. doi: 10.1016/j.rser.2022.112357

[8] Yao Y, Lian Z, Liu W, Shen Q. Experimental study on physiological responses and thermal comfort under various ambient temperatures. Physiol Behav. 2008;93(1–2):310–321. doi: 10.1016/j.physbeh.2007.09.012 17936860

[9] Yao Y, Lian Z, Liu W, Jiang C, Liu Y, Lu H. Heart rate variation and electroencephalograph–the potential physiological factors for thermal comfort study. Indoor Air. 2009;19(2):93–101. doi: 10.1111/j.1600-0668.2008.00565.x 19348034

[10] Wu M, Li H, Qi H. Using electroencephalogram to continuously discriminate feelings of personal thermal comfort between uncomfortably hot and comfortable environments. Indoor Air. 2020;30(3):534–543. doi: 10.1111/ina.12644 31943395

[11] Wu M, Qi H. Using passive BCI to online control the air conditioner for obtaining the individual specific thermal comfort. Proceedings of the 2019 41st Annual International Conference of the IEEE Engineering in Medicine and Biology Society (EMBC); 2019 Jul 23–27; Berlin, Germany. pp. 3139–3142. doi: 10.1109/EMBC.2019.8856497 31946553

[12] Shan X, Yang E-H, Zhou J, Chang VW-C. Human-building interaction under various indoor temperatures through neural-signal electroencephalogram (EEG) methods. Build Environ. 2018;129:46–53. doi: 10.1016/j.buildenv.2017.12.004

[13] Shan X, Yang E-H. Supervised machine learning of thermal comfort under different indoor temperatures using EEG measurements. Energy Build. 2020;225:110305. doi: 10.1016/j.enbuild.2020.110305

[14] Okamoto T, Tamura K, Miyamoto N, Tanaka S, Futaeda T. Physiological activity in calm thermal indoor environments. Sci Rep. 2017;7:11519. doi: 10.1038/s41598-017-11755-3 28912456 PMC5599655

[15] Tamura K, Matsumoto S, Tseng YH, Kobayashi T, Miwa J, Miyazawa K, et al. Physiological and subjective comfort evaluation under different airflow directions in a cooling environment. PLoS One. 2021;16(4):e0249235. doi: 10.1371/journal.pone.0249235 33852598 PMC8046250

[16] Tamura K, Matsumoto S, Tseng YH, Kobayashi T, Miwa J, Miyazawa K, et al. Physiological comfort evaluation under different airflow directions in a heating environment. J Physiol Anthropol. 2022;41:16. doi: 10.1186/s40101-022-00289-x 35428365 PMC9012013

[17] Raheel A, Anwar SM, Majid M. Emotion recognition in response to traditional and tactile enhanced multimedia using electroencephalography. Multimed Tools Appl. 2019;78:13971–13985. doi: 10.1007/s11042-018-6907-3

[18] Rolls ET, Grabenhorst F, Parris BA. Warm pleasant feelings in the brain. Neuroimage. 2008;41(4):1504–1513. doi: 10.1016/j.neuroimage.2008.03.005 18468458

[19] Delorme A, Makeig S. EEGLAB: An open source toolbox for analysis of single-trial EEG dynamics including independent component analysis. J Neurosci Methods. 2004;134(1):9–21. doi: 10.1016/j.jneumeth.2003.10.009 15102499

[20] Pascual-Marqui RD. Discrete, 3D distributed, linear imaging methods of electric neuronal activity. Part 1: exact, zero error localization. 2007 [cited 11 Aug 2022]. doi: 10.48550/arxiv.0710.3341

[21] Pascual-Marqui RD. Theory of the EEG inverse problem. In: Tong S, Thakor NV, editors. Quantitative EEG analysis: Methods and clinical applications. Artech House; 2009. pp. 121–140.

[22] Pascual-Marqui RD, Lehmann D, Koukkou M, Kochi K, Anderer P, Saletu B, et al. Assessing interactions in the brain with exact low-resolution electromagnetic tomography. Philos Trans R Soc A. 2011;369:3768–3784. doi: 10.1098/rsta.2011.0081 21893527

[23] Gramfort A, Luessi M, Larson E, Engemann DA, Strohmeier D, Brodbeck C, et al. MEG and EEG data analysis with MNE-Python. Front Neurosci. 2013;7:267. doi: 10.3389/fnins.2013.00267 24431986 PMC3872725

[24] Gramfort A, Luessi M, Larson E, Engemann DA, Strohmeier D, Brodbeck C, et al. MNE software for processing MEG and EEG data. Neuroimage. 2014;86:446–460. doi: 10.1016/j.neuroimage.2013.10.027 24161808 PMC3930851

[25] Welch P. The use of fast Fourier transform for the estimation of power spectra: A method based on time averaging over short, modified periodograms. IEEE Trans Audio Electroacoust. 1967;15(2):70–73. doi: 10.1109/TAU.1967.1161901

[26] Desikan RS, Ségonne F, Fischl B, Quinn BT, Dickerson BC, Blacker D, et al. An automated labeling system for subdividing the human cerebral cortex on MRI scans into gyral based regions of interest. Neuroimage. 2006;31(3):968–980. doi: 10.1016/j.neuroimage.2006.01.021 16530430

[27] Loshchilov I, Hutter F. Decoupled weight decay regularization. Proceedings of the International Conference on Learning Representations; 2019 May 6–9; New Orleans, Louisiana, USA. Available: https://openreview.net/forum?id=Bkg6RiCqY7

[28] Pedregosa F, Varoquaux G, Gramfort A, Michel V, Thirion B, Grisel O, et al. Scikit-learn: Machine learning in Python. J Mach Learn Res. 2011;12:2825–2830. Available: http://jmlr.org/papers/v12/pedregosa11a.html

[29] Abadi M, Agarwal A, Barham P, Brevdo E, Chen Z, Citro C, et al. TensorFlow: Large-scale machine learning on heterogeneous systems. 2015 [cited 11 Aug 2022]. Available from: https://www.tensorflow.org/

[30] Bergstra J, Bardenet R, Bengio Y, Kégl B. Algorithms for hyper-parameter optimization. In: Shawe-Taylor J, Zemel R, Bartlett P, Pereira F, Weinberger KQ, editors. Advances in Neural Information Processing Systems. Curran Associates, Inc.; 2011. Available: https://proceedings.neurips.cc/paper/2011/file/86e8f7ab32cfd12577bc2619bc635690-Paper.pdf

[31] Akiba T, Sano S, Yanase T, Ohta T, Koyama M. Optuna: A next-generation hyperparameter optimization framework. Proceedings of the 25th ACM SIGKDD International Conference on Knowledge Discovery & Data Mining; 2019 Aug 4–8; Anchorage, Alaska, USA. New York, NY, USA: Association for Computing Machinery; 2019. pp. 2623–2631. doi: 10.1145/3292500.3330701

[32] Holm S. A simple sequentially rejective multiple test procedure. Scand J Stat. 1979;6(2):65–70. Available: http://www.jstor.org/stable/4615733

[33] Phan KL, Wager T, Taylor SF, Liberzon I. Functional neuroanatomy of emotion: A meta-analysis of emotion activation studies in PET and fMRI. Neuroimage. 2002;16(2):331–348. doi: 10.1006/nimg.2002.1087 12030820

[34] Kanosue K, Sadato N, Okada T, Yoda T, Nakai S, Yoshida K, et al. Brain activation during whole body cooling in humans studied with functional magnetic resonance imaging. Neurosci Lett. 2002;329(2):157–160. doi: 10.1016/s0304-3940(02)00621-3 12165401

[35] Grabenhorst F, Rolls ET, Parris BA. From affective value to decision-making in the prefrontal cortex. Eur J Neurosci. 2008;28(9):1930–1939. doi: 10.1111/j.1460-9568.2008.06489.x 18973606

[36] Grabenhorst F, D’Souza AA, Parris BA, Rolls ET, Passingham RE. A common neural scale for the subjective pleasantness of different primary rewards. Neuroimage. 2010;51(3):1265–1274. doi: 10.1016/j.neuroimage.2010.03.043 20332031

[37] Farrell MJ, Johnson J, McAllen R, Zamarripa F, Denton DA, Fox PT, et al. Brain activation associated with ratings of the hedonic component of thermal sensation during whole-body warming and cooling. J Therm Biol. 2011;36(1):57–63. doi: 10.1016/j.jtherbio.2010.11.003

[38] Aizawa Y, Harada T, Nakata H, Tsunakawa M, Sadato N, Nagashima K. Assessment of brain mechanisms involved in the processes of thermal sensation, pleasantness/unpleasantness, and evaluation. IBRO Reports. 2019;6:54–63. doi: 10.1016/j.ibror.2019.01.003 30656240 PMC6329283

[39] Gohel B, Lim S, Kim M-Y, Kwon H, Kim K. Dynamic pattern decoding of source-reconstructed MEG or EEG data: Perspective of multivariate pattern analysis and signal leakage. Comput Biol Med. 2018;93:106–116. doi: 10.1016/j.compbiomed.2017.12.020 29291534

[40] Sato M, Yamashita O, Sato M, Miyawaki Y. Information spreading by a combination of MEG source estimation and multivariate pattern classification. PLoS One. 2018;13(6):e0198806. doi: 10.1371/journal.pone.0198806 29912968 PMC6005563

[41] Raheel A, Majid M, Alnowami M, Anwar SM. Physiological sensors based emotion recognition while experiencing tactile enhanced multimedia. Sensors (Basal). 2020;20(14):4037. doi: 10.3390/s20144037 32708056 PMC7411620

